# Fractional Flow Reserve or Coronary Flow Reserve for the Assessment of Myocardial Perfusion: Implications of FFR as an Imperfect Reference Standard for Myocardial Ischemia

**DOI:** 10.1007/s11886-018-1017-4

**Published:** 2018-07-26

**Authors:** Valérie E. Stegehuis, Gilbert W. Wijntjens, Jan J. Piek, Tim P. van de Hoef

**Affiliations:** Amsterdam UMC, University of Amsterdam, Department of Cardiology, Heart Centre, Meibergdreef 9, Amsterdam, The Netherlands

**Keywords:** Fractional flow reserve, Coronary flow reserve, Instantaneous wave-free ratio, Coronary physiology, Ischemic heart disease

## Abstract

**Purpose of Review:**

Accumulating evidence exists for the value of coronary physiology for clinical decision-making in ischemic heart disease (IHD). The most frequently used pressure-derived index to assess stenosis severity, the fractional flow reserve (FFR), has long been considered the gold standard for this purpose, despite the fact that the FFR assesses solely epicardial stenosis severity and aims to estimate coronary flow impairment in the coronary circulation. The coronary flow reserve (CFR) directly assesses coronary blood flow in the coronary circulation, including both the epicardial coronary artery and the coronary microvasculature, but is nowadays less established than FFR. It is now recognized that both tools may provide insight into the pathophysiological substrate of ischemic heart disease, and that particularly combined FFR and CFR measurements provide a comprehensive insight into the multilevel involvement of IHD. This review discusses the diagnostic and prognostic characteristics, as well as future implications of combined assessment of FFR and CFR pressure and flow measurements as parameters for inducible ischemia.

**Recent Findings:**

FFR and CFR disagree in up to 40% of all cases, giving rise to fundamental questions regarding the role of FFR in contemporary ischemic heart disease management, and implying a renewed approach in clinical management of these patients using combined coronary pressure and flow measurement to allow appropriate identification of patients at risk for cardiovascular events.

**Summary:**

This review emphasizes the value of comprehensive coronary physiology measurements in assessing the pathophysiological substrate of IHD, and the importance of acknowledging the broad spectrum of epicardial and microcirculatory involvement in IHD. Increasing interest and large clinical trials are expected to further strengthen the potential of advanced coronary physiology in interventional cardiology, consequently inducing reconsideration of current clinical guidelines.

## Introduction

It is widely recognized that coronary angiography (CAG) alone is inadequate in assessing functional coronary artery stenosis severity, and, consequently, in objectively determining the need for revascularization [1, 2]. The fractional flow reserve (FFR) has emerged as an important addition to coronary angiography for clinical decision-making in ischemic heart disease (IHD) [3, 4]. FFR-guided percutaneous coronary intervention (PCI) leads to effective alleviation of anginal complaints and similar low rates of adverse events, while reducing the number of revascularization procedures [1]. Consequently, FFR-guided revascularization was documented to be cost-effective compared with angiographic guidance alone [5, 6]. Moreover, identifying stenoses which may benefit from revascularization is important, as revascularization of non-ischemia generating stenoses might be unnecessary and harmful [7]. However, it is usually neglected that FFR is a pressure-derived estimation of coronary flow impairment, and is not the same as direct measures of coronary flow and flow reserve. Since the myocardium thrives on coronary flow, and not on perfusion pressure, coronary flow plays a more important role in maintaining myocardial function [8]. Coronary flow-based evaluation for the coronary circulation can, among other techniques, be performed using the coronary flow reserve (CFR), which represents the available vasodilator reserve capacity of the coronary circulation. CFR has shown a consistent strong prognostic value for adverse cardiac events, and seems to dominate risk stratification when FFR and CFR are assessed together [9••]. Nonetheless, mainly on practical grounds, FFR is currently used as the standard physiological tool in clinical practice. Moreover, its position in clinical practice guidelines has attracted investigators to use this tool as a reference standard in diagnostic studies. Yet, it is widely documented that FFR has distinct limitations with respect to its diagnostic value to detect coronary flow impairment, and its prognostic value derived from large randomized clinical trial data seems to be suboptimal. The aim of this review is to elaborate the basic physiology underlying concepts like FFR and CFR, and to discuss the inherent limitations for both clinical practice and scientific endeavors.

## Principles of Coronary Pressure and Flow

To accurately interpret clinical coronary physiology and physiological indices in contemporary clinical practice, substantial knowledge of the physiological coronary pressure and flow relationship both in absence and presence of a stenosis is required.

## Coronary Autoregulation in the Healthy Coronary Circulation

**Fig. 1:**
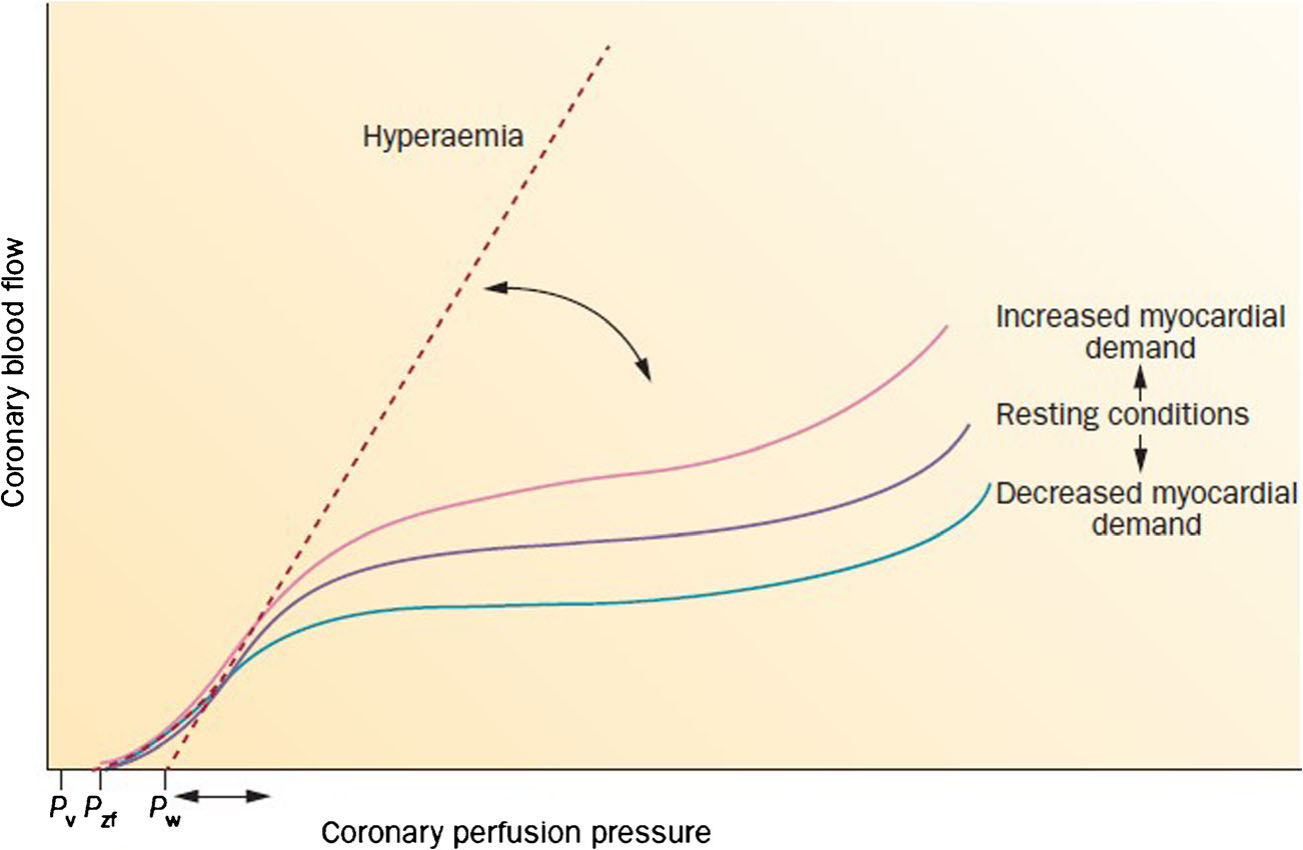
Pressure and flow relationship in resting conditions, during exercise and during hyperemia. When myocardial demand increases, a parallel shift occurs in coronary blood flow, whereas coronary flow and pressure have an incremental-linear relationship under hyperemia conditions. Two concepts, the metabolic adaptation and the coronary autoregulation, are important determinants of coronary flow during increased myocardial demand. In the presence of small vessel disease or diminished left ventricular function, a parallel shift in coronary flow occurs (curved arrow). The coronay wedge pressure (*P*_*w*)_ is the pressure-flow gradient extrapolated from the pressure-flow relationship during maximal vasodilation, whereas the zero-flow intercept (*P*_zf_) is marginally higher than the venous pressure (*P*_*v*_). Reproduced from van de Hoef TP et al., Nature Reviews Cardiology. 2013;10(8):439–52(64), with permission [11]

Coronary autoregulation describes the basic ability of the coronary circulation to adapt to changes in perfusion pressure or myocardial demand. Put simply, since the myocardium thrives on coronary flow to maintain its function, the coronary autoregulation process aims to maintain coronary flow at a level that meets myocardial demand by regulating vasodilator tone of the coronary resistance vessels. By such adaptive vasodilation or vasoconstriction, coronary flow in autoregulated (resting) conditions is largely independent of perfusion pressure at the normal clinical range of pressures [10]. This is illustrated by the plateau in the resting pressure-flow relationship in Fig. 1. With changes in myocardial demand, for example with exercise, coronary autoregulations also maintains coronary flow at a level that meets the increase in myocardial demand. This is illustrated by the parallel shift in the coronary pressure-flow relationship in Fig. 1 [12, 13]. At complete vasodilation, as is aimed for by the induction of pharmacological coronary hyperemia during coronary physiology studies, coronary autoregulation is eliminated. Since the compensatory mechanisms are then abolished, coronary flow depends on perfusion pressure. The relationship between coronary pressure and flow at complete vasodilation is, however, not proportional as it has a non-zero pressure intercept. In reality, the relationship between coronary pressure and flow at maximal hyperemia is incremental-linear in the physiological range of perfusion pressures (Fig. 1). This is important because it means that pressure cannot simply be used to estimate coronary flow, even during the condition of maximal coronary hyperemia.

## Coronary Pressure and Flow in the Presence of a Stenosis

### Compensatory Vasodilation

With worsening of epicardial coronary artery diameter reduction due to coronary artery disease, coronary flow to the distal microcirculation is progressively attenuated. Compensatory vasodilation of the coronary resistance vessels aims to maintain myocardial perfusion at a level that meets myocardial demand until all vasodilatory reserve is exhausted [14], and myocardial ischemia and its clinical sequelae occur [15]. Coronary flow remains stable in the presence of stenoses up to 50% diameter stenosis. Clinical studies have documented that maximal coronary flow becomes attenuated as soon as epicardial diameter reduction amounts to approximately 50%, whereas resting coronary flow becomes attenuated once a stenosis reached 80% diameter reduction [16, 17].

### Stenosis Physiology

**Fig. 2:**
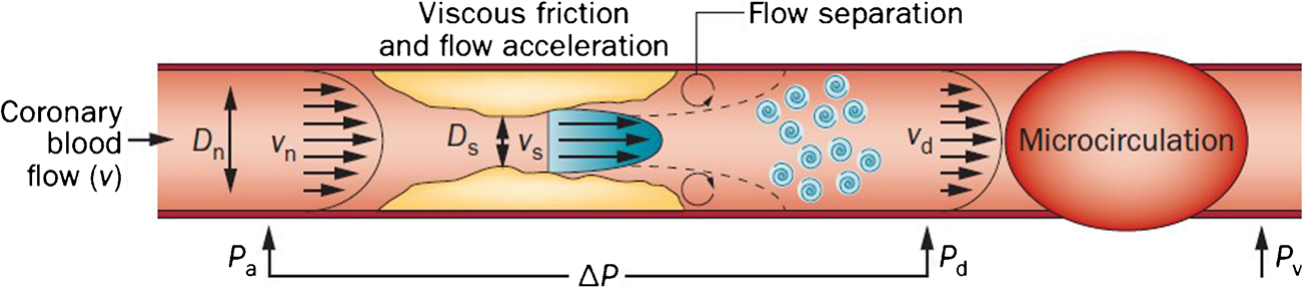
Pressure and flow across a stenosis. The pressure gradient across a stenosis is determined by the sum of viscous and separation losses. Flow separation and the formation of eddies prevent complete pressure recovery at the exit. Measurement of intracoronary hemodynamics includes proximal perfusion pressure (*P*_*a*_), coronary pressure and flow velocity distal to the stenosis (*P*_*d*_ and *V*_*d*_, respectively), and the venous pressure (*P*_*v*_), which is usually assumed to be negligible. Δ*P* is the difference between Pd and Pa. Normal diameter (reproduced from van de Hoef TP et al., Nature Reviews Cardiology. 2013;10(8):439–52(64), with permission) [11]) (*D*_*n*_), stenosis diameter (*D*_*s*_), proximal velocity (*V*_*n*_), and stenosis velocity (*V*_*s*_) are indicated. Adapted from van de Hoef TP et al., Nature Reviews Cardiology. 2013;10(8):439–52(64), with permission [11]

Focal coronary artery disease results in a reduction of the diameter of the coronary artery, which leads to pressure loss across the coronary artery due to the effects of viscous friction, convective acceleration, and flow separation. Pressure loss due to viscous friction occurs according to Poiseuille’s law, as the pressure across the stenosis attempts to overcome the friction between the two vessel walls. Viscous friction losses increase linearly with an increase in coronary flow through the stenosis. The convective flow acceleration as a result from the reduced diameter of the coronary artery leads to the conversion of pressure to kinetic energy loss according to Bernoulli’s law [18]. Separation of flow at the stenosis exit leads to the swirling of blood, also called “eddies,” leading to further loss of kinetic energy, which is not fully recovered once flow patterns have recovered. These pressure lossess according to Bernoulli’s law increase quadratically with an increase in flow through the stenosis. This combination of viscous friction, acceleration, and separation losses is unique for a given stenosis, and can be represented by pressure-drop flow velocity curves [19] (Fig. 2). The pressure-drop flow velocity relationship can be seen as the fingerprint of the stenosis.

## Basics of Fractional Flow Reserve: Hypothesis Versus Reality

In the early 1990s, the FFR was introduced as a pressure-derived estimate of coronary flow impairment due to the stenosis. It is calculated as the mean distal coronary pressure (Pd) divided by the mean aortic pressure (Pa) during maximal pharmacological vasodilation. The latter was induced by potent vasodilators such as adenosine, regadenoson, papaverine, or dipyridamole of which adenosine is recommended in daily clinical practice due to its short half-time and availability [20]. The theoretical framework of FFR is based on the basic assumption that the relationship between coronary pressure and flow is linear and proportional during maximal hyperemia. In this hypothetical scenario, distal coronary pressure would represent coronary flow in the stenosed coronary artery, whereas mean aortic pressure would represent the coronary blood flow in the coronary artery when no stenosis is present. Thereby, the FFR would represent the proportion of blood flow available to the distal myocardium relative to what is available should the stenosis not have been present [21–23].

Importantly, as noted previously, the actual relationship between coronary pressure and flow during maximal vasodilation is incremental linear. Thus, at best, coronary pressure may provide an estimate of coronary flow. Importantly, the actual slope of the pressure-flow relationship during coronary hyperemia is subject to many factors [21, 24], which leads to uncertainty with respect to the accuracy with which FFR is able to estimate coronary flow impairment: it may be close in some, but it may be vastly incorrect in others.

### Diagnostic and Clinical Validation of FFR

FFR has been validated against several non-invasive ischemia tests to determine its optimal cutoff value for inducible myocardial ischemia in patients with stable CAD [25]. The first cutoff value for FFR was determined at 0.66 for inducible myocardial ischemia determined with exercise stress testing [26], which later was increased to a FFR of 0.75, based on a combination of exercise stress tests, dobutamine stress echo, and myocardial perfusion imaging. In this study by Pijls et al., the FFR cutoff value of 0.75 was associated with 97% diagnostic accuracy for non-invasively determined ischemia-inducing stenoses [2]. Following these relatively small studies, a multitude of ischemia validation studies have been performed, where there overall optimal cutoff value of FFR was 0.75, with a diagnostic accuracy of 81% for non-invasively assessed myocardial ischemia. Diagnostic accuracy of FFR was highest in single-vessel disease, and, more importantly, validated solely in patients with stable CAD [27]. Following the initial documentation of a 0.75 FFR cutoff value for myocardial ischemia, this cutoff value was used in the first clinical validation study for FFR, the DEFER trial [7], which concluded that deferral of revascularization for FFR value ≥ 0.75 in patients with stable CAD is not associated with an increased risk of MACE [28]. The subsequent randomized FAME trials, however, used a higher FFR cutoff value of 0.80, so called clinical threshold, in order to minimize the number of hemodynamically significant stenoses deferred from revascularization. The first of these large clinical trials, FAME I, evaluated the clinical performance of FFR-guided PCI versus angiography-guided PCI using this 0.80 FFR cutoff value. The long-term results of the FAME trial have documented that FFR-guided PCI leads to equivalent long-term clinical outcomes compared with angiographic guidance, albeit with more swift alleviation of angina complaints while reducing the number of revascularization procedures required [25, 29]. The DEFER and FAME I and II trial findings have culminated in a class I level of evidence: A recommendation in the European Society of Cardiology (ESC) guidelines [30] and the ACC/AHA guidelines [31], where revascularization is advocated in all coronary stenoses with FFR ≤ 0.80. Nonetheless, expert opinion manuscripts from the founders of FFR support revascularization of stenoses with FFR < 0.75, and deferral of revascularization in stenoses with FFR > 0.80. Stenoses with FFR from 0.75 ranging to 0.80 pertain to the clinical FFR “gray zone,” where decision-making should be based on the results of other ischemic tests, as well as the individual risk-benefit profile of the patient. The latter results in both a clinical decision-making tool as well as a limitation of FFR, since individual variance in coronary physiology indices frequently occurs and thus impedes decision-making, especially in FFR values in or around the gray zone [32, 33].

Meanwhile, the results of the FAME II trial have shed new light on the clinical performance of FFR. FAME II randomized patients with at least one coronary artery with FFR ≤ 0.80 to optimal medical therapy alone or optimal medical therapy plus PCI. It was documented that PCI in addition to optimal medical therapy reduced the number of major adverse cardiac events through the first 2 years of follow-up. However, it is important to realize that the FAME II trial was prematurely halted because of a clear difference in the primary composite endpoint in favor of the PCI arm, thereby limiting the trial’s statistical power, and inducing a potential overestimation of effect size. Moreover, although significantly lower rates of adverse cardiac events were documented for PCI in addition to optimal medical therapy, it is important to realize that 60% of all patients with abnormal FFR values did not require revascularization, and 80% of patients with abnormal FFR values did not suffer from MACE throughout a 2-year follow-up period. Moreover, > 10% of vessels from patients in the reference group with normal FFR values, which were treated by optimal medical therapy alone, suffered MACE within the first 2 years of follow-up. Thus, the majority of FFR-positive vessels actually do not seem to be at risk for revascularization or hard clinical events, and a substantial proportion of FFR-negative vessels are adversely at risk for MACE within the first 2 years of follow-up. These results contradict the extremely high accuracy of FFR for inducible myocardial ischemia documented in the original multitest ischemic study. Subsequently, these results give rise to concerns regarding contemporary revascularization guidelines in which all FFR-positive stenoses are considered alike and eligible for coronary revascularization [34].

## Value of Non-Invasive Ischemia Detection in the FFR Era

The aforementioned non-invasive diagnostic modalities to identify inducible myocardial ischemia have been around for decades, and have been consistently documented to provide profound value for risk stratification purposes. Intrinsically, these techniques are subject to several influencing factors such as left ventricle hypertrophy, tachycardia and arrhythmias, and obesity, which influence diagnostic accuracy [35]. Moreover, imaging techniques are not uniformly able to discriminate between the role of epicardial and/or microvascular disease in the detection of myocardial ischemia, or to accurately determine the culprit coronary lesion, which can be cumbersome in patients with multivessel disease [36]. Despite these limitations, non-invasive imaging techniques often precede coronary physiological measurements, since determining the magnitude of myocardial ischemia is valuable before revascularization decision-making, particularly in the setting of borderline or equivocal FFR values, and is less invasive compared with immediate angiography [37]. Thereby, non-invasive imaging modalities constitute a valuable complement as these techniques are able to determine the anatomy and network of the larger coronary vessels and are able to designate a suspected ischemia inducing vessel or area. This clinical relevance of non-invasive imaging is frequently overlooked in clinical practice, where the majority of patients is referred to the cardiac catheterization laboratory without prior stress testing. This behavior likely follows from the clinical practice guidelines recommendation supporting the use of FFR as a surrogate of stress testing in patients in whom non-invasive stress testing was not performed. Thereby, the fundamental flaws in FFR theorem and its impact on diagnostic accuracy becomes more and more important in contemporary practice, since the absence of non-invasive stress testing means that clinical decision-making can only be performed on the basis of FFR assessment. This position of FFR in clinical guidelines and common clinical practice have also led investigators to use FFR as the reference test in the evaluation of novel non-invasive techniques for ischemia assessment. As can be concluded from basic coronary physiology characteristics and the physiological behavior of FFR, it is implausible that FFR should be considered a gold standard reference test.

FFR-CT, an emerging non-invasive tool to assess stenosis severity with imaging, shows reasonable diagnostic accuracy compared with invasively measured FFR. The FFR-CT is calculated by dedicated software, and with a FFR cutoff value of ≤ 0.8, FFR-CT results in a sensitivity of 78% and specificity of 87%, compared with FFR [38, 39]. However, for the evaluation of this novel technique, the FFR is yet again used as the gold standard. FFR-CT has not been validated in large clinical trials, but this is the point of interest in ongoing research endeavors such as the Assessing Diagnostic Value of Non-invasive FFRCT in Coronary Care (ADVANCE) trial (Clinicaltrials.gov NCT02499679). Apart from FFR-CT, many non-invasive techniques have traveled the path of diagnostic accuracy comparisons using FFR as the gold standard reference test. However, since FFR does not have the diagnostic (it was validated against non-invasive testing itself), or clinical (the majority of FFR-positive stenosis do not require revascularization) characteristics to be used as a gold standard reference test, the findings of such diagnostic accuracy studies should be interpreted with care. It might be the case that a novel non-invasive test is actually more accurate and provides more prognostic value than FFR, but loses research interest because initial comparisons with FFR lead to adverse results. Potentially, more advanced and comprehensive invasive techniques should be employed to identify the true diagnostic accuracy of such techniques before definitive conclusions are drawn.

## CFR: the Recurrence of Coronary Flow as an Indicator of Myocardial Ischemia

The coronary flow reserve and its value in assessing the coronary blood flow from both epicardial as well as the coronary microcirculation has been well studied [35, 40–42]. CFR is an index which is the ratio of coronary blood during maximal vasodilation divided by coronary blood flow during resting conditions [43]. CFR can be measured by either a temperature-sensitive guide wire applying the coronary thermodilution technique or a Doppler sensor equipped guide wire measuring Doppler flow velocity [44, 45]. Coronary thermodilution measurements require brisk saline injections during resting and hyperemic conditions to calculate CFR based on the average mean transit time of three saline boluses. Although it is considered correlate well to absolute coronary flow in research settings [46], this method is prone to measurement errors due to the sensitivity to the saline injections, and due to the fact that the rapid saline injections may disturb coronary hemodynamics, which is specifically deceptive in capturing basal flow values. The latter, however, is only identified when specific care is taken to obtain reasonably correlating mean transit times before calculation of CFR. The Doppler technique evidently provide Doppler flow velocity values, but also allows to track real-time the phasic flow velocity signal providing both additional information on the functional status of the coronary circulation, allows more advance physiology techniques to be employed, and provides direct feedback with regard to signal quality. It is, however, subject to operator’s experience and proper positioning of the guide wire in order to obtain a reliable flow signal [47]. Moreover, whereas coronary thermodilution-derived mean transit times reflect absolute coronary flow, and are therefore influenced by the amount of myocardial mass subtended by the stenosis, coronary flow velocity is intrinsically corrected for perfused myocardial mass by the laws of normalized wall shear stress [48–50].

Early experimental work of Smalling et al. has elegantly documented the dominant importance of coronary flow over coronary perfusion pressure for maintaining myocardial function. In his experimental study in dogs, he documented that coronary perfusion pressure could be reduced to values now considered equivalent to an FFR around 0.4 without affecting myocardial function as long as coronary flow was maintained [51]. The diagnostic characteristics of CFR itself have been extensively evaluated against non-invasive ischemia testing, similar to what was performed for FFR. Interestingly, the diagnostic accuracy of CFR for inducible myocardial ischemia on non-invasive stress testing was 81%, at an optimal cutoff value of 1.9, exactly the same as the accuracy for FFR [27, 35, 52]. Moreover, numerous non-randomized studies evaluated the prognostic value of CFR, where CFR was consistently documented to be strongly associated with clinical outcomes regardless of the modality used: the lower the CFR is, the higher the event rate is [53–55].

Several factors, however, have hampered the adoption of coronary flow and CFR assessment in clinical practice. The technical aspects discussed previously importantly have limited widespread adoption due to a lack of expertise with the techniques, and the associated increase in procedural time. Second, despite the numerous studies documenting its prognostic value, CFR is intrinsically sensitive to changes in hemodynamic conditions [21]. These aspects in comparison with the technical ease of coronary pressure measurements latter have contributed to the adoption of FFR and lack of CFR assessment in clinical practice.

However, as noted, coronary flow unequivocally plays a dominant role in cardiac function, and thus, routine coronary flow assessment could prove of great importance in clinical practice. Although the development of coronary flow techniques stagnated over the last years, recent research in the field of coronary flow and flow reserve led to novel understanding of the multilevel involvement of the coronary circulation in IHD [56, 57••], and has reinvigorated interest in coronary flow technology. To date, the Doppler guide wire is undergoing significant developments to improve its clinical use and feasibility, and the novel guide wire is highly anticipated. With more feasible assessment of coronary flow measurements, however, the time has come to address important questions regarding combined FFR and CFR measurements, for example, the occurrence, meaning, and optimal treatment of stenoses with disagreement between FFR and CFR.

## Combined Pressure and Flow Measurements in IHD: Stronger Together?

IHD is nowadays recognized to originate from a multilevel involvement of the coronary circulation. When CFR and FFR are assessed in combination, more detailed insight into the pathophysiological substrate of IHD can be obtained. Most easily, CFR and FFR can be interpreted according to their clinically applied cutoff values. Even though diagnostic accuracy for inducible myocardial ischemia is notably equivalent for both tools [27], disagreement between the two occurs in 30–40% of cases [57••]. Such disagreement has now been documented to illustrate distinct pathophysiology in IHD evaluation. A normal CFR and abnormal FFR—also termed non-flow-limiting coronary artery disease—characterize coronary stenoses that have normal to high coronary flow. Due to the occurrence of high coronary flow upon induction of maximal hyperemia, these stenosis impart high-pressure losses merely due to the fact that high trans-stenotic flows induce large pressure loss, as was discussed previously. These stenoses were documented to have benign long-term prognosis, and are like optimally managed medically. Potentially, the presence of a high number of non-flow-limiting stenosis among patients with abnormal FFR value in FAME II can explain the relatively benign prognosis in patients in FAME managed by medical therapy alone. Second, the occurrence of an abnormal CFR with normal FFR might characterize two pathophysiological substrates. It may reflect dominant microvascular disease where the vasodilator reserve capacity is hampered solely, or dominant diffuse epicardial disease where the occurrence of significant pressure drops is less likely compared with focal epicardial disease. These stenoses impart a particularly high risk for adverse events, and their optimal management remains to be elucidated [58, 59]. However, the clinical relevance of discordance between FFR and CFR, although physiologically plausible, has been established in retrospective studies only [52, 57••, 60•, 61]. The prospective evaluation of the prognostic relevance of FFR and CFR discordance is subject of the ongoing DEFINE FLOW study (NCT02328820).

### Flow-Derived Analysis of IHD: Putting Flow First

**Fig. 3:**
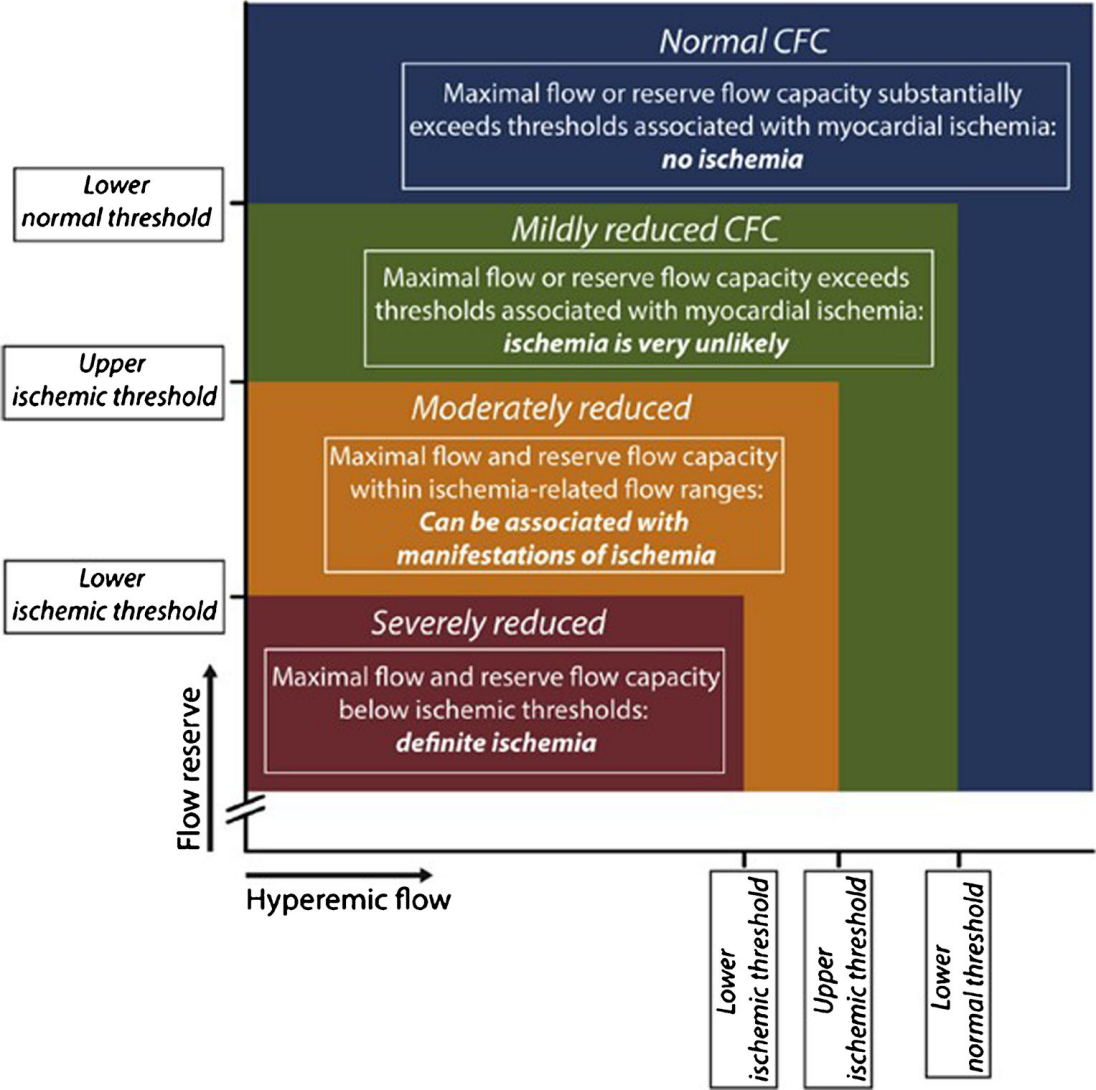
The principle of the Coronary Flow Capacity. Etc. Since coronary flow reserve (CFR) equals hyperemic to baseline average peak flow velocity (hAPV), a two-dimensional map of CFR versus hAPV comprehensively describes the invasive flow characteristics of the coronary vasculature under investigation. Within this concept, four clinically meaningful categories are defined (coded with different colors in the graph) based on well-validated invasive CFR cutoff values and the corresponding hAPV percentiles. Adapted from van de Hoef TP et al., JACC Cardiovascular Interventions. 2015;8(13):1670–80, with permission from Elsevier [52]

Considering the dominant role of coronary flow in myocardial function and clinical outcomes in IHD, a flow-based approach towards its management may be beneficial. However, as previously discussed, several limitations may apply to the use of CFR. These limitations led to the introduction of a novel concept which focuses solely on coronary flow and may overcome some of the limitations associated with the use of CFR: the coronary flow capacity (CFC) concept. This is a concept which stratifies coronary lesions on the basis of the combination of both maximal coronary flow and CFR values (Fig. 3) [52]. The CFC concept is based on the assumption that myocardial ischemia originates when both maximal coronary flow and the reserve capacity of the coronary circulation are below ischemic thresholds, and that such myocardial ischemia is unlikely once CFR or maximal flow is among normal values. The CFC calculated with invasive Doppler flow velocity values was documented to provide better risk stratification for MACE over CFR alone in patients with stable IHD and intermediate coronary stenoses. Moreover, with emerging non-invasive imaging to assess myocardial blood flow with PET, MRI, and CT, it is important to realize that CFC is intrinsically independent of the modality used to measure coronary flow parameters, and therefore has future potential for early detection IHD [62].

## Vasodilator-Free Assessment of Stenosis Severity

The instantaneous wave-free ratio (iFR) and resting Pd/Pa are two non-hyperemic pressure-derived indices which have been introduced to simplify coronary physiology assessment. Whereas resting Pd/Pa is calculated as the ratio of mean distal coronary pressure to mean aortic pressure across the whole cardiac cycle, iFR is calculated as the same ratio of the distal coronary pressure (Pd) divided by the aortic pressure (Pa) but restricted to the wave-free period, a distinct period during the diastole when coronary resistance is intrinsically low. Both iFR and Pd/Pa have similar high diagnostic accuracy for detecting myocardial ischemia compared with FFR [63]. Moreover, iFR-guided coronary intervention was recently proven non-inferior in terms of MACE rate during 1-year follow-up compared with FFR-guided coronary intervention in two large randomized clinical trials [64, 65]. Pd/Pa has not been evaluated in clinical outcome trials. Since iFR and Pd/Pa can be assessed without the use of vasodilators, they are not associated with the side effects associated with FFR measurements such as a total AV block, chest discomfort, or dyspnea. Additionally, iFR has distinct advantages in the evaluation of serial coronary stenoses. FFR is less favorable for this purpose due to the effect of stenosis interplay. In hyperemic conditions, coronary flow is reduced by a stenosis as soon as it reaches 50% diameter stenosis. This means that a relatively mild proximal stenosis hampers flow across the distal stenosis and vice versa. Since the magnitude of flow through the stenosis determines the pressure drop, and thus the FFR value, such interplay affects the FFR values of the individual stenosis. Hence, FFR does not allow to evaluate serial stenosis solitarily. In resting conditions, coronary flow is reduced by stenosis only when diameter stenosis exceeds 80–85%. Hence, stenosis interplay is much less likely in resting conditions. Consequently, iFR was documented to allow the assessment of individual contribution of stenosis to the over impairment in iFR, and, moreover, to allow prediction of the result of PCI in terms of iFR gain [66].

## Implications for (Future) Clinical Research

Coronary pressure-derived physiological indices are popular due to their simplicity, but do not fully address the complexity of CAD, which requires a more in detailed assessment of coronary physiology using both pressure- and flow-derived indices. Thus, the focus of future of coronary physiology may shift from pressure-derived indices to flow-derived indices, if more robust clinical data become available to further establish the diagnostic value of flow-derived indices in assessing stenosis severity and IHD. The latter requires simplification of current flow technology to improve practical application of these valuable physiology indices and make implementation less cumbersome.

## Conclusion

Although FFR has yielded an important progress in the diagnosis of obstructive coronary artery disease as a pressure-derived estimate of blood flow impairment, it has substantial intrinsic limitations that originate from the fact that FFR represents only an estimate of direct measures of coronary flow impairment from which it was derived. This is also illustrated by its prognostic characteristics: in the recent FAME II trial, the dominant proportion of FFR-positive stenoses did not require coronary revascularization. These considerations question the use of FFR as a gold standard reference test for the development of novel techniques for ischemia testing. With the documentation of a complex multilevel involvement of the coronary circulation in IHD, and the suboptimal performance of FFR-guided intervention, it is no longer tenable to delay the introduction of more comprehensive diagnostic strategies that aim to directly identify perfusion impairment, both for clinical decision-making and clinical research endeavors.
